# The double-edged sword of internet use in China’s aging population: thresholds, mediation and digital health policy

**DOI:** 10.3389/fpubh.2025.1643510

**Published:** 2025-07-16

**Authors:** Yirong Li, Jianguang Cai, Haiyong Yu

**Affiliations:** School of Physical Education, Hunan University of Science and Technology, Xiangtan, Hunan, China

**Keywords:** China’s aging population, internet use, health, double-edged sword, physical activity

## Abstract

**Background:**

Amid global aging and digital transformation, the dual effects of Internet use on health—its potential benefits and risks—remain a subject of debate. This study investigates the threshold effects of Internet use, the mediating role of physical activity, and the implications for digital health policy in China, revealing a compensation-vulnerability paradox among disadvantaged groups.

**Methods:**

The data were drawn from the 2020 China Family Panel Studies (CFPS), a nationally representative sample comprising 7,308 adults aged ≥45. We conducted multivariable regression with robustness checks and bootstrap mediation analysis. Nonlinear sensitivity was tested via U-shaped relationship validation and threshold effect identification.

**Results:**

Moderate Internet use (below 4.8 h/day) confers health benefits, but excessive use leads to adverse outcomes. Physical activity serves as a partial mediator in this relationship, exhibiting nonlinear mediation dynamics. Although the current average of 1.8 h/day of use internet is well below the harmful threshold, structural digital exclusion creates a compensation-vulnerability paradox: older and less-educated groups derive greater marginal health benefits from moderate use but face higher risks of overuse due to limited digital literacy and physiological constraints.

**Conclusion:**

Internet use offers conditional health benefits for older adults, contingent on usage thresholds and mediated by physical activity. Policymakers must balance targeted digital inclusion with safeguards against overuse, particularly for vulnerable groups. These findings highlight the need for time-bound digital health interventions and literacy programs to address the paradox of technological compensation and vulnerability, informing digital health policy in aging societies.

## 1 Introduction

Aging constitutes a continuous biological process characterized by progressive physiological decline (1), evolving family dynamics, and shifting social roles (2), exposing middle-aged and older adults to heightened risks of disability, chronic disease, loneliness, and depression. According to the communique on the Development of China’s Aging Cause in 2022, the number of older people aged 60 and above in China has reached 280 million, accounting for 19.8% of the total population (3), indicating that China has entered a deeply aging society. The degree of global aging is increasing, and health promotion must be included in any active response to the aging population.

The digital revolution has transformed social production and lifestyles. The rapid adoption of the Internet presents both opportunities and challenges for aging populations. In this study, Internet use is defined as using mobile devices or computers to access the Internet. Existing literature has highlighted a paradoxical relationship between Internet use and health among older adults. Many scholars argue that moderate Internet use can enhance the physical and mental health of the older adults. This is attributed to several factors, including the optimization of information dissemination (4), the acquisition of social support (5), the enhancement of a sense of gain (6) and the improvement of the medical service system (7). Conversely, some studies have shown that excessive Internet use have some negative effects on health of older adults. Addictive behaviors such as being engrossed in online social networks and short videos can reduce low-intensity physical activity time (8) and increase the risk of chronic diseases (9). Meanwhile, less time interacting with others in person is likely to undermine psychological well-being and social skills while increasing loneliness and depressive symptoms (10).

However, how to distinguish between moderate and excessive Internet use remains unclear. This paradoxical relationship between Internet use and health highlights three critical research gaps that directly address China’s aging-digital nexus. First, the duration threshold at which Internet use transitions from beneficial to detrimental remains undefined. Second, the mediating role of physical activity between Internet use and health status lacks empirical validation. Third, current policy frameworks fail to account for this nonlinearity when promoting digital inclusion for seniors. Our study leverages China Family Panel Study data to systematically examine this double-edged sword phenomenon through: (a) Identifying inflection points in Internet use duration that predict health status reversals, (b) Decipher physical activity’s mediating role, and (c) Developing tiered digital health policy recommendations based on identified thresholds. These findings will equip policymakers to maximize the benefits of technology while mitigating risks for China’s aging population.

## 2 Literature review and research hypothesis

### 2.1 Internet use and health of middle-aged and older adults

In recent years, scholars have conducted a series of studies on the impact of Internet use on the health of middle-aged and older people, yielding contradictory conclusions that Internet use can improve or damage health.

One is based on the network gain effect, which advocates that the Internet use can have a positive impact on the health status of older people (11, 12). According to the technology empowerment idea, some experts deem that the usage of the Internet offers distinct advantages in disease prevention and management. Due to its features such as low cost, high convenience, good accessibility, timely update and availability of detailed information, Internet can help older adults obtain relevant information on disease diagnosis and health promotion strategies (13–15), to realize the treatment of disease and prevention and the fitness of health in both hands. Another is based on the social network theory, some scholars believe that the Internet can break the physical limitations of time and space in traditional social modes and expand individual social networks (16). As a new type of social therapy, the Internet can provide more social contact and interaction for older adults and help them realize re-socialization, thus effectively alleviating loneliness and depression (17, 18).

The other viewpoint based on the negative impacts of the Internet, which holds that Internet use has no bearing on the health status of older people (19) and may even harm them (20). According to the presence substitution theory, some research suggest that using the Internet for social connection will reduce offline interpersonal engagement and communication while also reducing physical activity time (21). Individuals with lower levels of education are more likely to use the Internet for gaming and social interaction, which makes them more prone to over-reliance on the Internet (22). And the resulting Internet addiction can further reduce social participation and shrink social networks, leading to increased life stress and negative emotions (23). Some scholars also deem that older adults who have little or isolated social interaction in real life are more likely to use social networking sites frequently, and superficial communication in online social communication may reduce or lose social interaction in the real sense (24). There are also studies based on the social disengagement theory that older adults are physically and mentally fragile not suitable to play too many social roles and should withdraw from society at an appropriate time (25). Compared with those who do not go online, older adults who go online show fewer social roles and higher levels of disengagement, thus adapting better to later life and having higher life satisfaction (26).

The two opposing viewpoints are paradoxical in both theory and actual evidence, suggesting that the influence of Internet use on the health of middle-aged and older persons is not always favorable and may even be detrimental. Perhaps differences in the propensity to use the Internet and duration of use are major elements leading to this discrepancy (27). Internet use can promote health, however, there may be a duration threshold, and when the threshold is exceeded, Internet use can be detrimental to physical and mental health. To clarify the relationship between Internet use and health as well as explain the above contradictory opinions, this paper proposes the following hypotheses:

*H*1: Internet use demonstrates limited positive effects on the health status of middle-aged and older adults.

*H*2: Moderate Internet use can improve health, while excessive Internet use can be detrimental to health.

### 2.2 The mediating effect of physical activity

Physical activity represents one of the most potent non-pharmacological interventions for health enhancement. Extensive evidence confirms that scientifically guided, moderate physical activity effectively alleviates social isolation, enhances physiological resilience, prevents chronic diseases, and fosters holistic well-being in middle-aged and older adults (28, 29). Nevertheless, scholarly perspectives diverge regarding the relationship between Internet use and physical activity.

A prevailing consensus indicates that internet adoption significantly increases physical activity frequency among aging populations. The Internet enables knowledge acquisition about physical activity science, facilitates personalized training regimens, and optimizes movement techniques—collectively enhancing workout efficacy and adherence (30, 31). The proliferation of digital health applications further provides scenario-specific customization, elevating motivation and extending physical activity duration through tailored interventions (32, 33).

Conversely, substantial research demonstrates Internet use’s inhibitory effects on physical activity. Based on the time displacement effect, scholars contend that since both Internet use and physical activities occur during leisure hours, excessive online engagement displaces physical activity within individuals’ finite discretionary time, leading to predictable declines in physical activity levels (34). Algorithmically amplified content may also trigger behavioral addiction, disproportionately reducing movement time and precipitating cervical or lumbar pathologies and mood disturbances (35).

While academia acknowledges Internet use’s bidirectional influence on physical activity, mechanistic consensus remains elusive. Synthesizing evidence on the internet-activity-health nexus, this study proposes the hypothesis:

*H*3: Physical activity plays a mediating role between Internet use and health status.

*H*4: Internet use influences physical activity through a threshold mechanism: moderate Internet use promotes physical activity and enhances health status, whereas excessive Internet use diminishes physical activity and impairs health status.

## 3 Data and methods

### 3.1 Data sources

This study utilized 2020 survey data from the China Family Panel Studies (CFPS), a nationally representative longitudinal survey established by the Institute of Social Science Survey of Peking University. Designed to capture societal changes across economic, demographic, educational and health domains, CFPS employs a multistage probability-proportional-to-size (PPS) sampling design to ensure population representativeness. Our analytical sample restricted to middle-aged and older adults aged ≥45 years. After excluding participants under age 45 and cases with missing values on key variables, the final analytical cohort comprised 7,308 respondents.

### 3.2 Variable measurement

Dependent variables included physical and mental health. Physical health was operationalized using the Activities of Daily Living (ADL) scale, assessing independence across seven domains: independently outdoors, dining, kitchen tasks, public transportation use, shopping, hygiene and laundry. Each item employed binary coding (0 = unable, 1 = able), yielding a summative score ranging 0–7, where higher values indicate superior functional independence 1. Mental health was measured via the Center for Epidemiological Studies Depression Scale (CES-D), comprising six negatively phrased items (e.g., “I feel depressed”) and two reverse-scored positive items (e.g., “I feel happy”). Responses were coded on a 4-point frequency scale (0 = 5–7 days, 3 ≦1 day), with total scores spanning 0–24; elevated scores reflect fewer depressive symptoms and enhanced psychological well-being.

Independent variables included Internet use (binary: 1 = users, 0 = non-users) and daily Internet use duration (continuous: 0–24 h). To test curvilinear relationships, both the linear and quadratic terms of Internet use duration were incorporated into the regression models.

Frequency, duration and intensity of physical activity were examined as mediating variables. The operationalization comprised: (a) Physical activity frequency: Self-reported engagement over the previous 12 months, measured on an 8-point ordinal scale ranging from 0 (Never) to 7 (Twice daily or more). (b) Physical activity duration: Average time spent per session, quantified in minutes (range: 0–300). (c) Physical activity intensity: Perceived exertion during activities, assessed via a 4-point scale (0 = No exertion, 1 = Low, 2 = Moderate, 3 = High).

To minimize potential bias from excluded variables in the statistical model, pertinent variables impacting the health were incorporated as control variables. Demographic factors encompassed gender, age and marital status; socioeconomic factors comprised educational attainment, living in urban or rural areas and financial status; while lifestyle habits involved smoking and drinking, consuming protein, fruits and vegetables and sleep duration.

### 3.3 Modeling design

To elucidate the effects of Internet use on the health of middle-aged and older individuals, the following benchmark regression model has been constructed (Equation 1):


(1) $$\;{\text{Health}}_{i}=\beta_{0}+\beta_{1}{\text{Internet}}_{i}+\beta_{2}{\text{Time}}_{i}+\beta_{3}\mathrm{Tim}e_{i}^{2}+\beta_{4}X_{i}+\varepsilon_{i}\;$$

Among them, ${\text{health}}_{i}$ is the physical and mental health, Internet_i_ represents Internet usage, Time_i_ is the daily Internet usage time, Time_i_^2^ is the quadratic term of the daily Internet usage time, X_i_ is the control variable, $\varepsilon_{i}$ is the random interference term, and $\beta_{i}$ denotes the estimation coefficients.

To verify the mediating role of physical activity between Internet use and health, this paper employs a stepwise regression model to examine the intermediary effect of physical activity. The following is the construction process of the stepwise regression model (Equations 2–4):


(2) $${\text{Health}}_{i}=\alpha_{0}+\alpha_{1}{\text{Internet}}_{i}+\alpha_{2}{\text{Time}}_{i}+\alpha_{3}\mathrm{Tim}e_{i}^{2}++\alpha_{4}X_{i}+\theta_{i}\;$$

(3) $${\mathrm{PA}}_{i}=\gamma_{0}+\gamma_{1}{\text{Internet}}_{i}+\gamma_{2}{\text{Time}}_{i}+\gamma_{3}\mathrm{Tim}e_{i}^{2}+\gamma_{4}X_{i}+\in_{i}$$

(4) $$\begin{array}{l} {\text{Health}}_{i}=\delta_{0}+\delta_{1}{\text{Internet}}_{i}+\delta_{2}{\text{Time}}_{i}+\delta_{3}\mathrm{Tim}e_{i}^{2}+\\ \delta_{4}{\mathrm{PA}}_{i}+\delta_{5}X_{i}+\mu_{i} \end{array}$$

PA_i_ is physical activity of middle-aged and older people, $\alpha_{i},$ $\gamma_{i}$ and $\delta_{i}$ are the estimation coefficients, $\theta_{i},$ $\in_{i}$ and $\mu_{i}$ are the random interference terms, the rest of the settings are the same as in Equation 1.

### 3.4 Methods of statistical analysis

Analyses were conducted using SPSS 27.0 and STATA 17.0. Initial descriptive statistics characterized sample distributions. Multiple linear regression models assessed associations between Internet use (binary status and continuous duration, including quadratic terms for nonlinearity) and health outcomes, with U-tests validating quadratic term significance. Robustness was confirmed via propensity score matching (PSM), binary logistic regression substitution and explanatory variable replacement. Mediation effects of physical activity (frequency/duration/intensity) were tested using Bootstrap-derived 95% confidence intervals (5,000 resamples) with complementary U-tests. Finally, the seemingly unrelated regression (SUR) grouped approach was used to conduct heterogeneity analysis.

## 4 Results

### 4.1 Descriptive statistics

The descriptive statistics for middle-aged and older adults are presented in Table 1. The findings are as follows: (1) Regarding Internet use, only 35.6% of the respondents are Internet users, with an average daily online duration of 1.82 h. This indicates that a substantial proportion of older adults do not use the Internet and that the usage duration is relatively low. (2) In terms of health, Internet users have higher average scores in ADL and CES-D (6.87 and 18.73, respectively) compared to non-users (6.52 and 18.07), suggesting that Internet users tend to have better physical and mental health. (3) As for physical activity, Internet users have higher frequency, duration, and intensity of physical activity than non-users, indicating that Internet users are more actively involved in physical activity. (4) The results of the control variables show that Internet users have an average age of 55.12 years, with 52.7% residing in urban areas and 61.8% having a high school education or above. Compared to non-users, Internet users are younger, more likely to live in urban areas, and have a higher level of education. There are no significant differences in economic status and gender distribution between the two groups.

**Table 1:** Variable assignment and descriptive statistics results (*N* = 7,308).

Variables	Description	Internet users (*n* = 2,605,35.6%)		Non-Internet users (*n* = 4,703,64.4%)	
		Number (%)	Mean (SD)	Number (%)	Mean (SD)
ADL	0–7	2,605	6.87 (0.61)	4,703	6.52 (1.17)
CES-D	0–24	2,605	18.73 (4.17)	4,703	18.07 (4.48)
Duration of Internet use	Hours/day	2,605	1.82 (1.886)	4,703	0
Frequency of PA	1–7	2,605	1.96 (2.64)	4,703	1.25 (2.39)
Duration of PA	Minutes/times	2,605	21.19 (35.352)	4,703	11.60 (29.48)
Intensity of PA	0–3	2,605	0.69 (1.035)	4,701	0.35 (0.789)
Gender	Man = 1	1,426 (54.7)	0.55 (0.50)	2,293 (48.8)	0.49 (0.50)
	Woman = 0	1,179 (45.3)		2,410 (51.2)	
Age (2020)	45–59	2011 (77.2)	55.12 (7.75)	2,143 (45.6)	61.95 (9.45)
	60 and over	594 (22.8)		2,560 (54.4)	
Marital status	married = 1	2,382 (91.4)	0.91 (0.28)	4,079 (86.7)	0.87 (0.34)
	unmarried = 0	223 (8.6)		624 (13.3)	
Education level	Primary and below = 1	995 (38.2)	1.86 (0.78)	3,322 (70.6)	1.37 (0.62)
	junior = 2	967 (37.1)		1,035 (22.0)	
	Senior and above = 3	643 (24.7)		346 (7.4)	
Urban and rural residence	Urban = 1	1,374 (52.7)	0.53 (0.50)	1834 (39.0)	0.39 (0.49)
	Rural = 0	1,231 (47.3)		2,869 (61.0)	
Economic income level	lower = 1	763 (29.3)	1.92 (0.71)	1,193 (25.4)	2.08 (0.76)
	Medium = 2	1,281 (49.2)		1945 (41.4)	
	high = 3	561 (21.5)		1,565 (33.3)	
Smoking	Yes = 1	824 (31.6)	0.32 (0.47)	1,299 (27.6)	0.28 (0.45)
	No = 0	1781 (68.4)		3,404 (72.4)	
Drink alcohol	Yes = 1	435 (16.7)	0.17 (0.37)	700 (14.9)	0.15 (0.36)
	No = 0	2,170 (83.3)		4,003 (85.1)	
Protein	Yes = 1	2,234 (85.8)	0.86 (0.35)	3,580 (76.1)	0.76 (0.42)
	No = 0	371 (14.2)		1,123 (23.9)	
Vegetables and fruits	Yes = 1	2,567 (98.5)	0.99 (0.12)	4,574 (97.3)	0.97 (0.16)
	No = 0	38 (1.5)		129 (2.7)	
Sleep duration	Hours/day	707 (27.1)	7.19 (1.26)	1,155 (24.6)	7.39 (1.49)

ADL, Activities of daily living; CES-D, Center for Epidemiological Studies depression; PA, physical activity.

### 4.2 OLS regression

#### 4.2.1 Baseline regression

Multilinearity assays were performed before OLS regression analysis and variance inflation factor VIF estimates were less than 2 for all variables across the model, indicating that there are no multicollinearity problems and regression analysis can be performed. The regression results between Internet use and health are shown in Table 2. According to results, using the Internet can greatly enhance both mental and physical health, although the benefits are limited. Models 1 and 2 demonstrated significant positive associations between Internet use and health among middle-aged and older adults (ADL: *β* = 0.110; *p* < 0.01 and CES: *β* = 0.334; *p* < 0.01), confirming H1. Models 3 and 4 further investigate the association between Internet use duration and health. The findings indicate that there is an inverted U-shaped relationship between duration and health, implying that moderate Internet use can promote health up to a certain threshold. This validated H2’s proposition of optimal usage limits.

**Table 2:** Baseline OLS regression results.

Variables	Model 1: ADL	Model 2: CES-D	Model 3: ADL	Model 4: CES-D
Internet use	0.110*** (0.021)	0.344*** (0.115)		
Time			0.049*** (0.010)	0.162*** (0.064)
Time^2^			−0.004*** (0.001)	−0.017*** (0.008)
Gender	−0.002 (0.032)	0.772*** (0.123)	−0.002 (0.032)	0.772*** (0.125)
Age (2020)	−0.021*** (0.002)	0.029*** (0.006)	−0.021*** (0.002)	0.026*** (0.006)
Marital status	0.086 (0.037)	1.500*** (0.170)	0.086 (0.047)	1.500*** (0.170)
Education level	0.076*** (0.011)	0.392*** (0.048)	0.076*** (0.011)	0.400*** (0.048)
Urban and Rural residence	0.096*** (0.024)	0.980*** (0.102)	0.095*** (0.024)	0.984*** (0.102)
Economic income level	0.029*** (0.012)	0.519*** (0.047)	0.029*** (0.012)	0.517*** (0.047)
Smoking	0.047 (0.031)	−0.097 (0.129)	0.048 (0.031)	−0.093 (0.129)
Drink alcohol	0.109*** (0.029)	0.294** (0.136)	0.108*** (0.029)	0.293** (0.136)
Protein	0.117*** (0.032)	0.811*** (0.131)	0.117*** (0.032)	0.814*** (0.131)
Vegetables and fruits	0.509*** (0.120)	0.690** (0.336)	0.510*** (0.120)	0.691** (0.336)
Sleep duration	0.018 (0.027)	1.191*** (0.116)	0.018 (0.027)	1.187*** (0.116)
Constant	6.730*** (0.256)	2.458*** (0.982)	6.793*** (0.254)	2.708*** (0.972)
N	7,308	7,308	7,308	7,308
$R^{2}$	0.084	0.101	0.084	0.101

ADL, Activities of daily living; CES-D, Center for Epidemiological Studies depression; PA, physical activity. *p < 0.1; **p < 0.05; ***p < 0.01, SE, standard error in ().

When considering control variables, we observed significant gender differences in mental health, with males generally reporting better mental health than females. Consistent with Wurm’s findings (36), we noted that mental health tends to improve with age among older adults, even as physical health declines. Additionally, research supports the idea that having a spouse is linked to better mental health compared to being single (37). Other factors such as place of residence (rural vs. urban), education level, economic income, protein intake, and fruit and vegetable consumption all significantly impacted both physical and mental health. We also found that moderate alcohol consumption can enhance physical and mental well-being, echoing Marie’s perspective that alcohol can alleviate stress, offer enjoyment and relaxation, and yield positive short-term health benefits (38). Lastly, our research aligns with other studies (39, 40) in finding that middle-aged and older individuals who do not get enough sleep are more likely to experience depression-like symptoms.

#### 4.2.2 Inverted U relationship test

**Figure 1:**
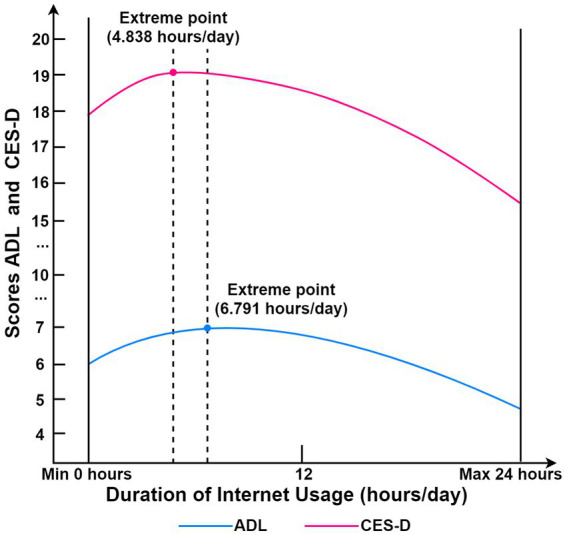
Inverted U-shaped relationship between internet use duration and health.

Haans pointed out that the existence of U-shaped relationships cannot be fully confirmed by the meaning of the quadratic term coefficients alone, so it is necessary to test U-shaped relationships (41). In Models 3 and 4, the relationship between Internet use life and health was tested in U-tests (Table 3; Figure 1). The extremal points for Internet surfing time in relation to ADL and CES-D are 6.791 and 4.838, respectively. The slopes for the intervals to the left of these points are greater than zero, while those to the right are less than zero. These slopes are statistically significant at the 1% level, thereby supporting H2, which posits that the impact of Internet use on the health of middle-aged and older individuals initially increases and then decreases. The degree to which Internet use affects physical and mental health varies, as do their respective thresholds.

**Table 3:** Results of U-test analyses 1.

Variables	ADL		CES-D	
	Lower limits	Upper limits	Lower limits	Upper limits
Interval	0	24	0	24
Slope	0.049	−0.0955	0.163	−0.506
T-value	5.100	−3.641	2.553	−1.962
*p*-value	0.000	0.000	0.001	0.025
Extreme points	6.791		4.838	

### 4.3 Robustness checks

#### 4.3.1 Exclude S relationships

To rule out the potential of an S-shaped association between the Internet use duration and health, this paper creates the cubic term ( ${\text{time}}^{3}$ ) of Internet use duration and inserts it into the regression equation. The results demonstrate that the regression coefficients of the cubic term ( ${\text{time}}^{3}$ ) of Internet use duration are not significant. However, the regression coefficients of the primary term ( time ) and the quadratic term ( ${\text{time}}^{2}$ ) are significant at the 1% level, which confirmed a stable inverted U-shaped association between Internet use duration and health.

#### 4.3.2 Replace the regression model

To further investigate the potential biases in model specification, this study employs a replacement regression approach by substituting the OLS model with a binary logistic model to assess the robustness of the findings. In this process, continuous values were transformed into binary variables. The coding guidelines for Activities of Daily Living (ADL) are as follows: zero for groups with low activity (scores 0–4) and one for those with high activity (scores 5–7). For the CES-D scale, groups with psychological depression are coded as zero (scores 0–15), while those with better mental health are coded as one (scores 16–24). Table 4 presents the results of the binary logistic regression analysis, which indicates the sustained positive impact of Internet use on health and confirms the inverted U-shaped relationship between usage duration and health. These results are consistent with the baseline regression findings and further reinforce the validity of the conclusions drawn.

**Table 4:** Binary logistic regression results.

Variables	Model 5: ADL	Model 6: CES-D	Model 7: ADL	Model 8: CES-D
Internet use	0.658*** (0.196)	0.165*** (0.068)		
Time			0.454*** (0.136)	0.100*** (0.040)
Time^2^			−0.028*** (0.011)	−0.009*** (0.004)
Controls	Yes	Yes	Yes	Yes
Constant	4.887*** (1.077)	−6.013*** (0.541)	4.976*** (1.076)	−5.930*** (0.537)
N	7,038	7,038	7,038	7,038
Pseudo $R^{2}$	0.128	0.062	0.128	0.062

ADL, Activities of daily living; CES-D, Center for Epidemiological Studies depression; PA, physical activity. *p < 0.1; **p < 0.05; ***p < 0.01, SE, standard error in ().

#### 4.3.3 Propensity score matching

Consider that using the Internet is a self-chosen habit impacted by personal qualities. To address the issue of selectivity bias, this research employs the propensity score matching technique (PSM) to conduct the robustness test, which complements the benchmark regression results. The equilibrium test of the samples revealed that the samples matched by different methods were well balanced, the standard deviation of each variable after matching was less than 5% and the variables with significant differences between the experimental group and the control group before matching were not significant after matching, and the self-selection bias was effectively alleviated, and the balance test passed, which met the requirements of the propensity score matching method.

In this study, we employed three matching approaches—K-nearest neighbor matching, radius matching, and kernel matching within calipers—to validate the impact of Internet use on health. The average treatment effect on the treated (ATT) for the entire sample is presented in Table 5. After eliminating the sample bias between the control and treatment groups, the ATT obtained is significant and relatively consistent across the different matching methods, which supports the robustness of our findings. Specifically, after accounting for potential confounding variables, the results align with those obtained in models 1 and 2, confirming that Internet use significantly promotes the physical and mental health of middle-aged and older adults.

**Table 5:** Propensity score match estimation results.

Estimation Methods		ADL			CES-D		
		ATT	Standard error	T-value	ATT	Standard error	T-value
Before matching		0.349	0.025	14.12***	0.658	0.107	6.16***
After matching	K neighbor matching within calipers	0.101	0.029	3.42***	0.303	0.149	2.03***
	Radius matching	0.107	0.031	3.50***	0.308	0.136	2.25***
	Nuclear matching	0.106	0.031	3.40***	0.312	0.138	2.26***

K neighbor matching in calipers, set K = 5, caliper value = 0.05, core matching specified bandwidth = 0.05, all others are default values. ATT, average treatment effect.

### 4.4 Mediation effect test

#### 4.4.1 Stepwise regression test

**Figure 2:**
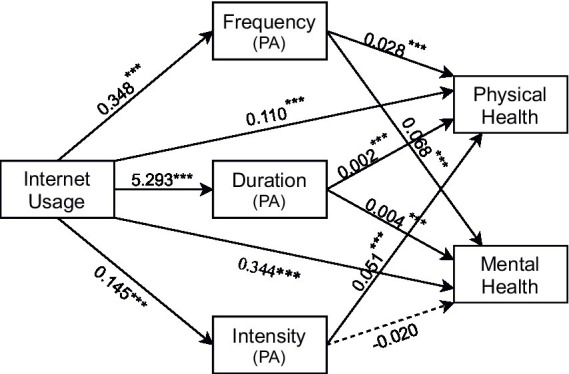
Multiple mediating effects model.

The study selected the frequency, duration, and intensity of physical activity as mediating variables to construct a multiple mediation model. The relevant variables were validated using stepwise linear regression and the Bootstrap mediation effect test method. As shown in Table 6, the regression coefficients of the core explanatory variables show that with the inclusion of the three indicators of physical activity, ${\text{time}}_{i}$ and ${\mathrm{PA}}_{i}$ are both significantly positive at the 1% level, and ${\text{time}}_{i}^{2}$ is significantly negative at the 1% level, except of the regression coefficients of physical activity intensity as a mediator variable in model 17, which are not significant. Thus, H3 was partially validated. The frequency and duration of physical activity significantly mediated the relationship between Internet use and both physical and mental health. Additionally, the intensity of physical activity significantly mediated the relationship between Internet use and physical health. These mediation effects are illustrated in Figure 2. Furthermore, an inverted U-shaped relationship was observed between Internet use duration and physical activity. Moderate Internet use can enhance the frequency, duration, and intensity of physical activity, while excessive use can have an inhibitory effect. Moreover, physical activity intensity did not mediate the relationship between Internet use and mental health, which aligns with the previous conclusion that different levels of physical activity intensity have varied effects on mental health (42).

**Table 6:** Multiple mediating regression results.

Variables	Frequency (PA)	ADL	CES-D	Duration (PA)	ADL	CES-D	Intensity (PA)	ADL	CES-D
	Model 9	Model 10	Model 11	Model 12	Model 13	Model 14	Model 15	Model 16	Model 17
Time	0.348*** (0.040)	0.039*** (0.010)	0.139*** (0.064)	5.293*** (0.551)	0.041*** (0.010)	0.142*** (0.064)	0.145*** (0.016)	0.042*** (0.010)	0.165** (0.064)
Time^2^	−0.023*** (0.004)	−0.003*** (0.001)	−0.015*** (0.008)	−0.371*** (0.058)	−0.003*** (0.001)	−0.015*** (0.008)	−0.010*** (0.002)	−0.003*** (0.001)	−0.017** (0.007)
Frequency (PA)		0.028*** (0.005)	0.068*** (0.021)						
Duration (PA)					0.002*** (0.001)	0.004*** (0.002)			
Intensity (PA)								0.051*** (0.110)	−0.020 (0.055)
Controls	Yes	Yes	Yes	Yes	Yes	Yes	Yes	Yes	Yes
N	7,038	7,038	7,038	7,038	7,038	7,038	7,038	7,038	7,038
$R^{2}$	0.115	0.088	0.102	0.077	0.086	0.102	0.079	0.086	0.101

ADL, Activities of daily living; CES-D, Center for Epidemiological Studies depression; PA, physical activity. *p < 0.1; **p < 0.05; ***p < 0.01, SE, standard error in ().

#### 4.4.2 Bootstrap mediation effect test

As shown in Table 7, bootstrap analyses (5,000 resamples) revealed significant indirect effects of physical activity in Models 18–22 (all *p* < 0.001; 95% CIs: [0.0038–0.2737], excluding zero), indicating that frequency and duration of physical activity partially mediated‌ the associations between Internet use and both physical and mental health. It means that the frequency, duration and intensity of physical activity played partial mediation effects between Internet use and health. In contrast, Model 23 showed nonsignificant mediation for physical activity intensity (*p* > 0.1; 95% CI: [−0.0184–0.2921]), confirming its lack of mediating role in the Internet-mental health relationship. These findings partially support H3, highlighting domain-specific mediation pathways.

**Table 7:** Bootstrap analyses of mediating effects.

Models	Type of effect	ADL			Models	CES-D		
		Effect size [95%CI]	(%)	Standard error		Effect size (95%CI)	(%)	Standard error
Model 18: Frequency	Indirect	0.010^***^[0.0059–0.0136]	20.41	0.002	Model 21: Frequency	0.024^***^[0.0085–0.0386]	14.72	0.008
	direct	0.039^***^[0.0199–0.0587]	79.59	0.010		0.139^***^[0.0042–0.2737]	85.28	0.069
Model 19: Duration	Indirect	0.009^***^[0.0047–0.0128]	18.36	0.002	Model 22: Duration	0.020^***^[0.0042–0.0368]	12.34	0.008
	direct	0.040^***^[0.0210–0.0597]	81.64	0.010		0.142^***^[0.2018–0.2638]	87.66	0.062
Model 20: Intensity	Indirect	0.008^***^[0.0038–0.0112]	16.00	0.002	Model 23: Intensity	−0.003 [−0.0184–0.0126]	-	0.008
	direct	0.042^***^[0.0221–0.0610]	84.00	0.010		0.165^***^[0.0387–0.2921]	-	0.065

CI, Confidence Interval. ADL, Activities of daily living; CES-D, Center for Epidemiological Studies depression; PA, physical activity. *p < 0.1; **p < 0.05; ***p < 0.01, SE, standard error in ().

#### 4.4.3 Inverted U relationship test

To further investigate the inverted U-shaped relationship between Internet use and physical activity among middle-aged and older people, this paper performed a U-test on the relationship between Internet use duration and physical activity (Models 9, 12 and 15), and the results are shown in Table 8. The results show that the turning points of the duration of Internet use and frequency, duration and intensity of physical activity are 7.141, 7.138 and 7.115, respectively. The slopes of the left side intervals are all >0 and the slopes of the right side intervals are all <0 and significant at the 1% level. The foregoing findings provide support for Hypothesis 4.

**Table 8:** Results of U-test analyses 2.

Variables	Frequency (PA)		Duration (PA)		Intensity (PA)	
	Lower bound	Upper bound	Lower bound	Upper bound	Lower bound	Upper bound
Interval	0	24	0	24	0	24
Slope	0.348	−0.583	5.293	−9.537	0.145	−0.262
T-value	8.689	−4.057	9.610	−5.074	9.185	−4.589
P > |t|	0.000	0.000	0.000	0.000	0.000	0.000
extreme point	7.141		7.138		7.115	

### 4.5 Heterogeneity test

The aforementioned findings reported the influence of Internet use on the physical and mental health of middle-aged and older adults. However, these effects may vary across different groups. To examine the heterogeneity in the influence of Internet use on health across age and education categories, this research employed the Seemingly Unrelated Regression (SUR) approach, which allows for the identification of significant differences. As shown in Table 9, the inverse U-shaped relationship between Internet use duration and health is more pronounced among individuals over 60 years old and those with primary school education or below. These groups exhibit greater efficacy in health enhancement but also have a lower threshold for adverse effects.

**Table 9:** Results of heterogeneity test.

Variables	ADL					CES-D				
	Model 21:age		Model 22: Education level			Model 23:age		Model 24: Education level		
	45–59	≧60	Primary	Junior	Senior	45–60	≧60	Primary	Junior	Senior
Time	0.048^***^	0.111^***^	0.107^***^	0.049^***^	0.057^***^	0.100^*^	0.161^*^	0.249^***^	0.047	0.057^***^
	(0.010)	(0.029)	(0.022)	(0.016)	(0.021)	(0.059)	(0.098)	(0.086)	(0.080)	(0.021)
Time^2^	−0.003^***^	−0.009^***^	−0.009^***^	−0.003	−0.003^**^	−0.008	−0.017^*^	−0.021^**^	0.001	−0.003^**^
	(0.000)	(0.029)	(0.002)	(0.001)	(0.002)	(0.006)	(0.010)	(0.009)	(0.008)	(0.002)
Controls	Yes	Yes	Yes	Yes	Yes	Yes	Yes	Yes	Yes	Yes
Constant	5.533^***^	5.073^***^	5.117^***^	6.468^***^	6.828^***^	4.038^***^	4.686^***^	5.215^**^	6.230^***^	6.828^***^
	(0.252)	(0.379)	(0.296)	(0.330)	(0.449)	(1.202)	(1.272)	(1.171)	(1.541)	(0.449)
Coefficient difference	9.47^***^		10.58^***^			0.31		4.98^*^		
Extreme points	7.677	6.477	6.167	9.375	8.292	6.680	4.806	5.901	7.003	8.292
N	4,154	3,154	4,314	2002	992	4,154	3,154	4,314	2002	992
R^2^	0.041	0.046	0.064	0.033	0.045	0.089	0.113	0.078	0.077	0.045

ADL, Activities of daily living; CES-D, Center for Epidemiological Studies depression; PA, physical activity. *p < 0.1; **p < 0.05; ***p < 0.01, SE, standard error in ().

Firstly, the Coefficient of Variation between groups reveals that the values for ADL (CV_age=_9.47, CV_edu_ = 10.58, *p* < 0.001) were all more significant than those for CES-D (CV_age_ = 0.31, *p* > 0.1; CV_edu_ = 4.98, *p* < 0.1). This indicates that Internet use has a more pronounced effect on physical health, as evidenced by age and educational level differences. Secondly, the baseline regression results show that the coefficient of Internet use duration is larger and more significant for individuals over 60 years old $(\beta_{\mathrm{ADL}}=0.111,\beta_{\mathrm{ADL}}^{2}=-0.009,$ *p* < 0.001; $\beta_{\mathrm{CES}-D}=0.161,$ $\beta_{\mathrm{CES}-D}^{2}=-0.017,$ *p* < 0.05) and those with primary school education or below $(\beta_{\mathrm{ADL}}=0.107,\beta_{\mathrm{ADL}}^{2}=-0.009,$ *p* < 0.001; $\beta_{\mathrm{CES}-D}=0.249,$ $\beta_{\mathrm{CES}-D}^{2}=-0.021,$ , *p* < 0.001) compared to those aged 45–60 and those with junior high school education or above. The inverse U-shaped relationship is more evident in these groups, suggesting that older individuals and those with lower education levels derive greater health benefits from Internet use. This highlights the significant role of the Internet in improving health among vulnerable groups such as the older adults and those with low education. Thirdly, the distribution of extreme points across all models indicates that individuals over 60 years old ( ${\mathrm{EP}}_{\mathrm{ADL}}=6.477,{\mathrm{EP}}_{\mathrm{CES}-D}=4.806$ ) and those with primary school education or below ( ${\mathrm{EP}}_{\mathrm{ADL}}=6.167,{\mathrm{EP}}_{\mathrm{CES}-D}=5.901$ ) have lower extreme points compared to their younger and more educated counterparts. This illustrates that older individuals or those with lower education levels are more susceptible to digital addiction and have a lower threshold for negative health impacts.

## 5 Discussion

### 5.1 Principal findings

Based on the nationally representative data from the 2020 China Family Panel Survey (CFPS), this study investigates the relationship between Internet use and health among middle-aged and older populations in China through OLS regression, logistic regression, U tests and propensity score matching. Although existing research has confirmed the correlation between these two factors, the internal mechanisms remain underexplored within a systematic theoretical framework. To address this gap, we transcend traditional linear assumptions by integrating Internet enrichment effects and digital harm theory to examine their nonlinear relationship. Empirical results demonstrate that the health-promoting effects of Internet usage are constrained, with positive outcomes contingent upon moderate online engagement duration. Several important conclusions can be drawn from this study.

First, this study confirms an inverted U-shaped relationship between Internet use and the physical and mental health of middle-aged and older groups, revealing the dual mechanism of digital empowerment and harm. Moderate Internet use can significantly improve health by facilitating the acquisition of online health information (43), enhancing health behavior management (44) and strengthening social interaction (45). These findings support the technologically enabling pathway of the digital empower theory. However, when Internet use duration exceeds the 4.8-h threshold, it triggers the substitution effect of cognitive anxiety and sedentary behavior due to information overload (46), leading to diminishing or even reversed health returns. This aligns with the threshold effect mechanism of digital hazard theory (35, 47). Notably, mental health exhibits greater sensitivity to overuse, with earlier inflection points potentially attributable to this population’s limited information-filtering capacity during initial stages of technological adaptation. Emotional vulnerability to false health information further exacerbates anxiety-behavioral dependency cycles (12, 48). Currently, the average duration of daily Internet use among middle-aged and older groups is 1.8 hours, significantly below the 4.8-h negative-effect threshold, indicating that net health benefits of Internet use remain within a positive range.

Second, our analysis reveals that physical activity plays a partial mediating role between the Internet and health, which is consistent with the conclusion of previous studies that physical activity as a healthy lifestyle can mediate between digital life and healthy life (49), but the difference is that the pathway of physical activity is nonlinear. Moderate Internet use enhances physical activity frequency and duration among middle-aged and older adults through motor skill augmentation and socially driven physical activity motivation, thereby improving health outcomes (42, 50). Conversely, excessive online engagement beyond 7.1 h daily leads to screen-time encroachment, substantially suppressing physical activity and manifesting the adverse consequences of the time substitution hypothesis. It is worth noting that the mediating role of physical activity intensity on mental health remains unverified. This may relate to the physical activity preferences of older populations, who often enhance psychological well-being through socially embedded low-intensity activities such as walking and tai chi, rather than intensity-driven regimens (51, 52).

Additionally, the study’s grouped regression analysis demonstrates that the health-promoting potential of Internet use is particularly pronounced among older adults and low-education populations. However, these groups also exhibit lower negative-effect thresholds. This compensation-vulnerability paradox among disadvantaged groups can be interpreted through the interplay of resource substitution theory and digital exclusion theory. The resource substitution effect suggests that older adults and low-education individuals, constrained by limited offline health resources, increasingly rely on the Internet as compensatory health capital (53), thereby gaining greater marginal health benefits (54, 55). However, these groups simultaneously face a dual capacity deficit characterized by difficulties in accessing digital resources and digital education (56) and age-related physiological functional decline (57), rendering them more susceptible to overuse and entrenching a vulnerability trap. While digital technologies can act as compensatory tools to reduce health disparities in vulnerable populations, this necessitates age-appropriate design and regulated online time to mitigate risks of digital exclusion (58). These findings underscore the importance of aligning technological interventions with the specific needs and capabilities of disadvantaged groups to foster equitable health outcomes.

### 5.2 Policy recommendations

This paper proposes the following policy recommendations: (1) Implement tiered digital services for older adults. Implement tiered digital services for older adults. Customize service interfaces based on age and educational disparities, developing basic templates for older adult and low-education groups to simplify interaction processes, while designing advanced modes for middle and high-digital-literacy populations to precisely adapt to functional needs. (2) Establish a collaborative mechanism for intelligent supervision and literacy enhancement. Integrate user health data and behavioral profiles to create a dynamic usage threshold system, enabling real-time alerts and adaptive online time adjustments. Concurrently, prioritize critical digital literacy training focused on strengthening information discernment and time management skills, thereby mitigating overuse incentives. (3) Develop a smart fitness service ecosystem. Generate personalized low-intensity physical activity plans using physical health monitoring data, embed social incentive mechanisms to enhance physical activity adherence and cultivate proactive fitness awareness. This approach aims to integrate physical activity into the daily lives of middle-aged and older adults, fostering active aging and advancing healthy longevity. These recommendations align with the need for age-inclusive technological interventions and capacity-building strategies to balance digital empowerment with safeguards against overuse, ultimately supporting equitable health outcomes for vulnerable populations.

### 5.3 Limitations

Our study based on 2020 cross-sectional data is limited in establishing causality and temporal sequences due to endogeneity issues. Prior research (46, 59) with longitudinal panel data and fixed effects models has shown a causal link between Internet use and the health of older adults, providing empirical support for causality in our study. Second, while the study focuses on the impacts of Internet use and physical activity on the health of older adults, other factors such as intergenerational support (60), access to medical resources (61) and lifestyle patterns (62) also significantly influence health outcomes in this population and warrant further exploration. Third, given the rapid advancement of smart technologies and the deepening digital transformation in China, the digital engagement levels of older adults post-2020 have likely increased significantly. Future investigations could leverage updated datasets to conduct more nuanced analyses as supplementary data becomes available.

## 6 Conclusion

This study identifies an inverted U-shaped relationship between Internet use and health among middle-aged and older adults, with a critical threshold of 4.8 h of Internet use duration beyond which health risks emerge. This highlights the dual role of digital technology in health empowerment and harm. Physical activity mediates the relationship between Internet use and health, exhibiting nonlinear characteristics. The current average daily usage (1.8 h) remains below the risk threshold, suggesting untapped potential for digital health interventions. However, structural digital exclusion creates a compensation-vulnerability paradox: while older adult and low-educated groups derive higher marginal health benefits from moderate use, their limited digital literacy and physiological decline increase overuse risks, exacerbating health disparities. These findings emphasize the need for balanced policies to optimize digital health benefits while safeguarding vulnerable populations.

## Data Availability

The raw data underpinning the findings of this article will be provided by the authors without any restrictions. For individuals interested in accessing the raw CFPS data, please visit the official website: http://www.isss.pku.edu.cn/cfps/index.htm.
